# The added value of coupling anti-dsDNA and anti-chromatin antibodies in follow-up monitoring of systemic lupus erythematosus patients

**DOI:** 10.1016/j.jtauto.2025.100274

**Published:** 2025-01-22

**Authors:** Caroline Carlé, Françoise Fortenfant, Chloé Bost, Julie Belliere, Stanislas Faguer, Dominique Chauveau, Antoine Huart, David Ribes, Laurent Alric, Gregory Pugnet, Laurent Sailler, Yves Renaudineau

**Affiliations:** a Immunology Department Laboratory, Referral Medical Biology Laboratory, Institut Fédératif de Biologie, Toulouse University Hospital Center, France; b INFINITy, Toulouse Institute for Infectious and Inflammatory Diseases, INSERM U1291, CNRS U5051, University Toulouse III, Toulouse, France; c Department of Nephrology and Organ Transplantation, Referral Centre for Rare Kidney Diseases, University Hospital of Toulouse, INSERM U1297, Toulouse, France; d Internal Medicine, University Toulouse III, Toulouse, France

**Keywords:** anti-Chromatin, anti-dsDNA, Diagnostic, Flare, Follow-up, SLE

## Abstract

**Objective:**

The autoimmune response to chromatin (Chr) or one of the nucleosome components (double stranded (ds)DNA and histones) is typically associated with the development of systemic lupus erythematosus (SLE). Related autoantibodies (Ab) are heterogeneous and, among them, anti-dsDNA Ab are part of the classification criteria and recommended for monitoring SLE with regards to lupus flares and therapy responses. However, anti-dsDNA Ab biomarker performances are weak; therefore, coupling anti-dsDNA with anti-Chr Ab can be proposed, which is the aim of this study.

**Methods:**

In this single center study from 2009 to 2024, 269 SLE patients with follow-up information were retrospectively selected from a population of 646 individuals, including 325 SLE patients, who tested positive for anti-dsDNA and/or anti-Chr Ab (Bioplex 2200™). Bio-clinical information during follow-up were assessed at several time points through medical records in order to explore associations between the anti-dsDNA/Chr Ab profile with disease presentation, and anti-dsDNA/Chr Ab fluctuations with disease activity using clinical SLEDAI-2K, flares requiring specific treatment, and the therapeutic response.

**Results:**

At inclusion in the follow-up analysis, corresponding to diagnosis (116/269, 43.1 %) or flare (153/269, 56.9 %), SLE patients were subdivided into three serological groups: the double positive dsDNA/Chr group (DP+, 190/269: 70.6 %), followed by the single positive Chr group (SP-C+, 42/269: 15.6 %), and the single positive dsDNA group (SP-D+, 37/269: 13.8 %). The DP + group, which presented important anti-dsDNA/Ab variations during follow-up, was at risk to develop lupus nephritis (56.8 % versus 2.4 % in SP-C+ and 29.7 % in SP-D+ groups, p < 0.04) and serositis (30 % versus 9.5 % in SP-C+ group, *p* = 0.006). During follow-up, anti-dsDNA and Chr Ab levels in the SP-C+ and SP-D+ groups remained stable over time irrespective of disease activity, flares, and therapeutic response. Regarding the DP + group, disease activity was correlated with both anti-dsDNA (RmCorr = 0.46, p = 1.6x110-^91^) and anti-Chr (RmCorr = 0.38, p = 2.8x10-^60^) Ab levels, which can be used to predict flares. Following therapy introduction, Ab reduction occurred in all patients from the DP + group with a more pronounced effect reported in complete responders.

**Conclusion:**

coupling anti-dsDNA with anti-Chr Ab detection at disease initiation/flare allows definition of endotypes, which is useful to follow disease activity, predict lupus nephritis/serositis, and anticipate therapeutic response in the DP + group.

## Highlights

• There was added value in coupling anti-dsDNA/Chr antibody determination in SLE.
• The double positive dsDNA+/Chr+ (DP+) group was associated with a more severe disease.
• Ab levels in the DP + group can be used to monitor disease activity, flare, and therapy response.
• Lupus nephritis and serositis were rare events in the single positive Chr + group.

## Abbreviations:

Ab: autoantibodies
ANA: antinuclear Ab on HEp-2 cells
ANOVA: analysis of variance
AID/ID: autoimmune and inflammatory diseases
AUC: area under the curve
BCR: B cell receptor
BILAG: British isles lupus assessment group index
Chr: chromatin
CR: complete response
DP+: double positive anti-dsDNA/Chr Ab group
dsDNA: double-stranded DNA
DORIS: definition of remission in SLE
ENA: extractable nuclear Ab
IQ: interquartile
NR: non responder
PR: partial responder
RmCOrr: repeated-measure correlation
RNP: anti-ribonucleoprotein Abs
ROC: receiver operating curve
SLE: systemic lupus erythematosus
SLEDAI-2K: SLE disease activity score 2000
Sm: anti-Smith Abs
SP-C+: single positive anti-Chr Ab group
SP-D+: single positive anti-dsDNA Ab group
SSA/SSB: anti-sicca syndrome A/B Abs

## 1 Introduction

Systemic lupus erythematosus (SLE) is a chronic and multisystem autoimmune disease that requires long term monitoring in order to prevent organ damage and mortality. Risk factors for poor outcomes are related to persistently elevated levels of antinuclear autoantibodies (ANA), higher disease activity, numerous flares, cumulative usage of immunosuppressant drugs including glucocorticoids, and a proliferative lupus nephritis [1,2].

B-cell dysregulation is central in the pathophysiology of SLE as reflected by an altered B cell subset distribution, hyper/hypo gammaglobulinemia, a large panel of autoantibodies (Ab), complement consumption through the immunoglobulin-dependent classical pathway, and SLE control under B-cell targeting drugs[[3], [4], [5]]. More than 180 Ab have been reported in SLE, some have been incorporated in the SLE classification criteria (e.g. anti-double stranded (ds)DNA, anti-Smith (Sm), and anti-phospholipid Ab), and, among them, only anti-dsDNA Ab detection is considered in the follow-up explaining its inclusion in the SLE disease activity score (SLEDAI), and the other composite scores, which are used to monitor flares, relapses and/or therapeutic responses [[6], [7], [8]]. However, using anti-dsDNA Ab for the follow-up of SLE patients is debated due to the heterogeneity of the immune response to native dsDNA, which may affect the clinical value of the assays with important variations reported between them [9,10]. In such situations, additional biomarkers are mandatory, and one of them may be anti-chromatin (Chr) Ab. Indeed, dsDNA is part of the nucleosome that is the basic unit of chromatin, which consists of 145–147 pairs of DNA wrapped around an histone octamer. Anti-Chr Ab can appear up to 2 years before anti-dsDNA Ab, present better diagnostic clinical performance as compared to dsDNA Ab, but their utility during follow-up remained to be established [11,12].

To evaluate the effectiveness of combining anti-dsDNA with anti-Chr Ab to monitor follow-up in SLE patients, a two-step study was undertaken. Initially, 325 SLE patients were identified from those who tested positive for either anti-dsDNA or anti-Chr Ab. Subsequently, fluctuations in anti-dsDNA/Chr Ab levels among 269 SLE patients, representing a broad range of the disease manifestations, were evaluated longitudinally with regards to treatment effect, disease activity, and flare occurrences. For a more comprehensive analysis of these fluctuations, all patients underwent testing using the same technology (Bio-Plex 2200™) during their follow-up and were categorized into three serological groups: the double positive dsDNA group (DP+), which exhibited significant variations in anti-dsDNA/Chr Ab levels during follow-up as compared to the single positive anti-dsDNA (SP-D+) or anti-Chr (SP-C+) groups. Given the important Ab level variations that may arise during the disease trajectory, serological groups were established based on the initial occurrence corresponding to an active phase of the disease to minimize misclassification.

## 2 Material and methods

### 2.1 Patient selection

**Fig. 1:**
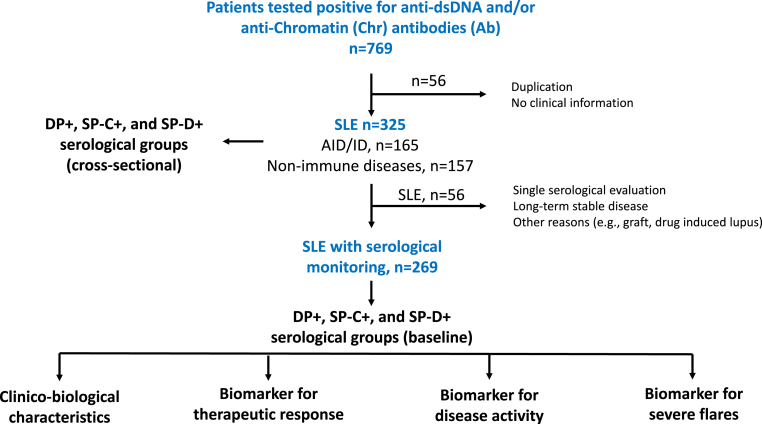
**Flowchart of study design.** A total of 647 patients who tested positive for either anti-dsDNA or anti-Chromatin (Chr) autoantibodies (Ab), along with clinical information, were initially selected from a broader population of 10,766 patients. These patients were subsequently categorized into double or single dsDNA/Chr Ab positive groups (DP+, SP-C+, and SP-D+) to evaluate their ability to qualitatively and quantitatively differentiate systemic lupus erythematosus (SLE) patients from those with non-SLE autoimmune/inflammatory diseases and other diseases. Observing that significant variations in anti-dsDNA/Chr Ab levels were associated with the DP + serological group among 269 SLE patients selected from the initial cohort, with serological monitoring extending over a period of up to 15 years, the 3 serological groups were redefined at baseline. Baseline corresponded to an active disease occurring at diagnosis or during a severe flare episode. Subsequently, clinico-biological characteristics of these serological groups were compared, as well as their efficacy as biomarkers for reflecting therapeutic response (baseline vs last-time point or first negative time point), disease activity fluctuation (across all follow-up time points), and severe flares (pre-flare vs flare time points).

To select a large panel of SLE patients for the analysis, a two-step retrospective selection was conducted as summarized in Fig. 1. From January to December 2023, a preliminary cohort of 647 patients, all of whom tested positive for either anti-dsDNA or anti-Chr Ab and possessed available clinical information, was selected from a broader population of 10,766 patients investigated for these Ab using the ANA Bio-Plex system (Bio-Plex 2200™, Bio-Rad, Hercules, CA) at the immunology tertiary laboratory of Toulouse University Hospital Center. Within this initial cohort, 325 individuals were diagnosed with SLE at various stages of the disease. Among them, 269 patients underwent longitudinal analysis concerning anti-dsDNA/Chr Ab over a period extending up to 15 years (2009–2024). Serological data were collected during routine follow-up consultations scheduled at intervals of 1, 3, 6 or 12 months based on the patient's disease activity and during episodes of relapse. Furthermore, information obtained from medical records, throughout the follow-up period, encompassed age, sex, disease duration, clinical manifestations, current treatments, flares necessitating specific treatment due to new or worsening clinical activity, and the clinical (c)SLEDAI-2K score excluding biological parameters such as anti-dsDNA Ab. All SLE patients satisfied the 2019 ACR/EULAR classification criteria [13], and when clinically indicated, a percutaneous kidney biopsy was performed to confirm the diagnosis of lupus nephritis [14].

In order to investigate the evolution of anti-dsDNA/Chr Ab throughout the follow-up period, several time points were analyzed: (i) the inclusion visit (time = 0), which was selected during an active phase of the disease corresponding to diagnosis (116/269, 43.1 %) or when not available to a flare (153/269, 56.9 %), aimed at defining the serological groups of anti-dsDNA/Chr Ab and their associated characteristics; (ii) the initial and final visits (time = 0 and 1) aimed at analyzing the progression of anti-dsDNA/Chr Ab level following the introduction/intensification of therapy at the inclusion visit. This analysis categorized the therapeutic response between T = 0 and T = 1 as follows: complete responders (those who achieved the 2021 DORIS criteria after first-line of treatment and without any subsequent flare); non-responders (defined as cSLEDAI ≥4 during follow-up); while others not achieving the DORIS criteria and/or having flares with therapy intensification during follow-up were categorized as partial or relapsing responders [15,16]; (iii) time to achieve negativity following therapy introduction/intensification from T = 0; (iv) All follow-up visits occurring between T = 0 and T = 1 were analyzed to explore correlation between anti-dsDNA/Chr Ab levels and disease activity; and (v) pre-flare and flare visits were assessed to evaluate variations in anti-dsDNA/Chr Ab levels associated with flare in a subset of SLE patients (n = 140).

The study was conducted according to the guidelines of the declaration of Helsinki. The participants were informed according to the French medical legislation, and the study was approved by the ethics committee in France (CPP) under the references RC31/21/0154 (Nephrogen) and 2021-A03236-35 (ESSAi).

### 2.2 Immunological parameters

The immunological panel for SLE-associated biomarkers collected at various time points during follow-up included in addition to the IgG anti-dsDNA and anti-Chr Ab when available: (i) the complement fractions C3 and C4 (Cobas 500®, Roche Diagnostics GmBH, Germany); (ii) ANA titer and staining using the ANA patterns classification on HEp-2 cells [17] (Kallestad HEp-2 cells, Bio-Rad, Hercules, USA); (iii) IgG anti-extractable nuclear (ENA) Ab against sicca syndrome (SS)A 52 kDa, SSA 60 kDa, SSB, Sm, Sm-ribonucleoprotein (RNP), RNP A, and RNP 68 kDa antigens plus IgG anti-ribosomal Ab (Bio-Plex 2200™, Bio-Rad, Hercules, CA); and (iv) IgG anti-cardiolipin (CL) and IgG anti-beta 2 glycoprotein I (β2-GPI, Bio-Plex 2200™, Bio-Rad, Hercules, CA) [[18], [19], [20]]. Cut-offs were fixed as recommended by the manufacturers (anti-dsDNA Ab ≥10 international units [IU]/mL, anti-ENA/-Ribosomal/-Chr Ab ≥ 1.0 arbitrary units [AU]mL, anti-CL/β2-GPI Ab > 20 U/mL, C3 low <0.72 g/L; andC4 low <0.11 g/L).

### 2.3 Statistical analysis

Quantitative data are presented as median and interquartile (IQ) at 25th-75th percentile and analyzed using non-parametric tests with Dunn's test applied for post-hoc multiple comparisons in analysis of variance (ANOVA, Kruskal-Wallis test) when necessary. Categorical data were analyzed using Fisher's exact test. The repeated measures correlation test (rmcorrShiny (shinyapps.io)) was applied to account for the dependent structure of the same patient over time between two parameters, and RmCorr values >±0.3 were considered significant. Receiver operating characteristic (ROC) curves were generated to determine the area under the curve (AUC). Kaplan–Meier curves and the log-rank (Mantel-Cox) test were used to assess time to achieve Ab negativity. The alpha risk was set to 0.05 and statistical analysis performed using GraphPad Prism 10.2 (La Jolla, CA).

## 3 Results

### 3.1 Anti-dsDNA/Chr Ab groups are helpful to discriminate SLE patients in a cross-sectional and preliminary analysis

**Fig. 2:**
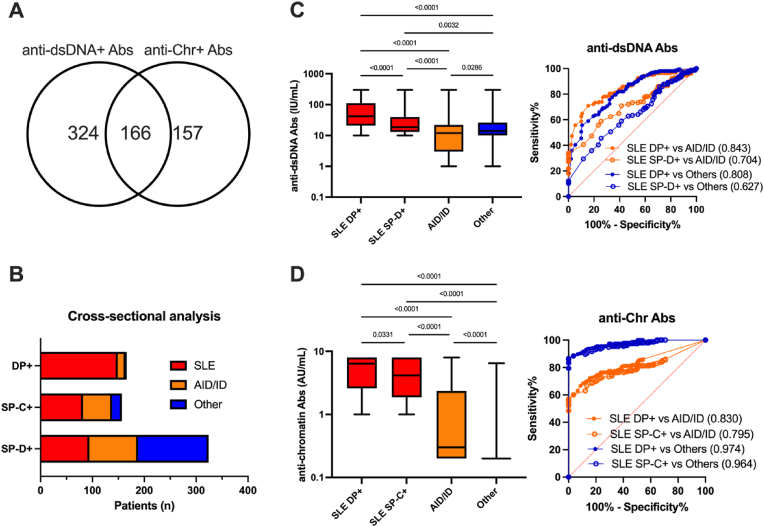
**Anti-dsDNA and anti-chromatin (Chr) serological groups are helpful to discriminate systemic lupus erythematosus (SLE) patients in a cross-sectional and preliminary analysis. A:** dsDNA/Chr group repartition using as a threshold ≥10 international units [IU]/mL for anti-dsDNA autoantibody (Ab) and ≥1.0 arbitrary units [AU]mL for anti-Chr Ab. **B:** Clinical distribution according to the serological groups: double positive anti-dsDNA/Chr group (DP+), single positive anti-Chr group (SP-C+), and single positive anti-dsDNA group (SP-D+). Clinical groups were defined as follows: SLE patients (red), non-SLE autoimmune and inflammatory diseases (AID/ID, orange), and other diseases (blue). **C:** Anti-dsDNA Ab levels according to the clinical groups and receiving operating characteristic (ROC) curves performed to compare anti-dsDNA Ab levels between SLE patients according to their serological group and patients with AID/ID (orange) or other diseases (blue). **D:** anti-Chr Ab levels according to the clinical groups. Area under the curve (AUC) and p values are reported (Kruskal-Wallis test with Dunn's post-hoc correction).

In 2023, 769/10,766 (7.1 %) patients tested positive for anti-dsDNA and/or anti-Chr Abs. Among them, and after excluding those without clinical information or duplication, 647 patients corresponding to 325 SLE patients at different stages of their disease, 165 patients with another autoimmune/inflammatory disease (AID/ID; e.g., mixed/undifferentiated connective tissue disease, rheumatoid arthritis, Sjögren's syndrome), and 157 patients with non-immune diseases (e.g. tumors, infections) were selected and subdivided according to their anti-dsDNA/Chr Ab profile in three serological groups (Fig. 2A/B): the single positive dsDNA group (SP-D+, 324/647: 50 %), followed by the double positive dsDNA/Chr group (DP+, 166/647: 25.7 %), and the single positive Chr group (SP-C+, 157/647: 24.3 %).

SLE patients were over-represented within the DP + group with 149/166 (89.8 %), followed by 82/157 (52.2 %) in the SP-C+ group, and 94/324 (29.0 %) in the SP-D+ group (see Table 1). AID/ID patients were more frequently found in the SP-C+ group (56/157, 35.7 %), while patients with other diseases predominated in the SP-D+ group (136/324, 42.0 %).

**Table 1:** Characteristics of the three anti-dsDNA/chromatin serological groups from the preliminary and cross-sectional analysis.

	DP+	SP-C+	SP-D+	*p* values DP + vs SP-C+	*p* values DP + vs SP-D+
Number	166/647 (25.7 %)	157/647 (24.3 %)	324/647 (50 %)		
F:M	147:19	129:28	217:107	0.114	<10^−4^
Age, median (IQ)	37y (30–51)	45.5y (35–61)	55.5y (41–70)	0.999	0.042
SLE	149/166 (89.8 %)	82/157 (52.2 %)	94/324 (29.0 %)	–	–
AID/ID	15/166 (9.0 %)	56/157 (35.7 %)	94/324 (29.0 %)	–	–
Other diseases	2/166 (1.2 %)	19/157 (12.1 %)	136/324 (42.0 %)	–	–
IgG anti-dsDNA, IU/mL	41.5 (21–111)	<10 IU/mL	16 (12–30)	–	<10^−15^
IgG anti-Chr, AU/mL	6.6 (2.5–8)	3.3 (1.3–8.0)	<1.0 AU/mL	2x10^−5^	–

Abbreviations: F: female; M: male; IQ interquartile; SLE: systemic lupus erythematosus; AID/ID: non-SLE autoimmune/inflammatory diseases; Chr: chromatin; DP+: double positive dsDNA/Chr group; SP-C+: single positive Chr group; SP-D+: single positive dsDNA group; y: years; IU/AU: international/arbitrary units. Statistical analysis: categorical data were analyzed using the Fisher's exact test (2x2), and quantitative data using the non-parametric Mann-Whitney test.

We found elevated levels of anti-dsDNA (p < 10^−4^) and anti-Chr (p = 0.03) Ab levels in the SLE-DP + group as compared to the corresponding SLE-SP + groups (Fig. 2C/D), allowing nevertheless to discriminate SLE-DP+ and SLE-SP + patients from AID/ID (0.704<AUC<0.843). Anti-Chr Abs best discriminate other diseases from the SLE-DP + group (AUC = 0.974, *p* < 10^−4^) and from the SLE SP-C+ group (AUC = 0.964, *p* < 10^−4^).

### 3.2 Anti-dsDNA/Chr Ab groups as biomarkers for disease severity

**Fig. 3:**
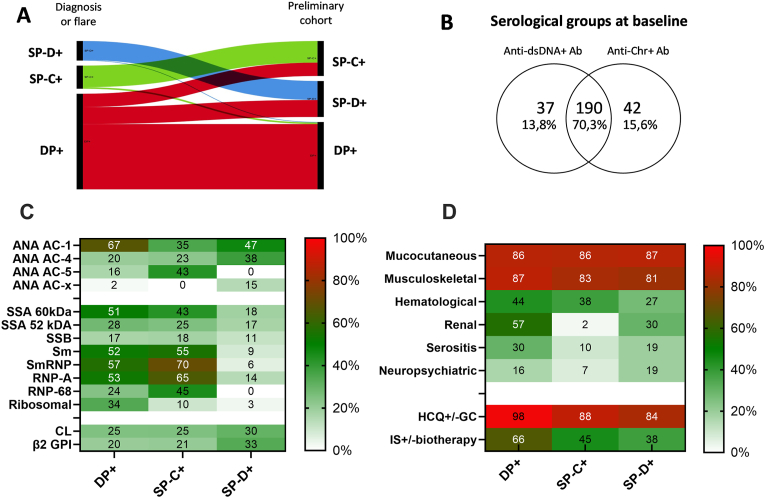
**Clinical and biological characteristics of the anti-dsDNA/Chromatin (Chr) serological groups in a retrospective cohort of 269 patients with SLE patients subjected to longitudinal serological monitoring. A:** Sankey diagram illustrates the evolution of the 3 groups: double positive for anti-dsDNA/Chr antibody (Ab, DP+), single positive for anti-Chr Ab (SP-C+), and single positive dsDNA Ab (SP-D+) group across two two-time points: the first one corresponding to the inclusion in an active phase of the disease (diagnosis or severe flare) and the second one corresponding to the cross-sectional and preliminary cohort assessment. **B:** Distribution of the dsDNA/Chr groups at baseline in the cohort (time = 0). **C:** Baseline biological characteristics. **D:** Clinical characteristics observed during follow-up, alongside medication usage at inclusion. For statistical analysis, refer to Table 2. Abbreviations: HCQ: hydroxychloroquine; GC: glucocorticoids; IS: immunosuppressant; DP+: anti-dsDNA and anti-Chr Ab double positive group; SP-D+: anti-dsDNA single positive group; dsDNA-/Chr-: anti-Chr single positive group; ANA: antinuclear antibody on HEp-2 cells; AC: anti-cellular (AC) patterns according to the ICAP initiative(12).

To assess the utility of combining anti-dsDNA with anti-Chr Ab for monitoring SLE, 269 SLE patients from the initial cohort were selected, all of whom underwent longitudinal follow-up with a median duration of 4.7 years (2.1–8.4). Serological data were collected at each visit, when available, with the inclusion visit (T = 0) corresponding to an active disease phase occurring at either diagnosis or during a flare. As illustrated in the Sankey diagram (Fig. 3A), which depicts the evolution of the 3 serological group from T = 0 to the preliminary analysis, 59/186 patients classified as DP+ in the longitudinal analysis have evolved to the SP-C+ (n = 33) and the SP-D+ (n = 26) groups. Conversely, only 4 patients comprising 3 SP-C+ and 1 SP-D+ at diagnosis exhibited seroconversion to DP+, coinciding with a flare occurring between 1.4 and 4.1 years following their SLE diagnosis. These 4 patients were subsequently reclassified into the DP + group, with their inclusion fixed at the time of seroconversion for subsequent analysis. From this analysis, we conclude that SP-C+ and SP-D+ groups exhibited stability over time, whereas the DP + group may present negativity for anti-dsDNA and/or anti-Chr Ab over time.

Next, clinico-biological differences in the follow-up cohort between the DP + group (190/269: 70.6 %), the SP-C+ (42/269: 15.6 %), and the SP-D+ (37/269: 13.8 %) groups were assed at inclusion as reported in Table 2 and Fig. 3B/C. The DP + group was characterized by: (i) a higher disease activity as compared to the SP-C+ group (p = 0.002); (ii) a more aggressive therapy introduction/intensification when considering both hydroxychloroquine ± glucocorticoids (0.002<*p* < 0.012) and immunosuppressant ± add-on biotherapies (*p* = 0.021 vs SP-C+ and *p* = 0.003 vs SP-D+ groups; (iii) a distinct ANA staining distribution on HEp-2 cells with AC-1 (homogeneous, 66.9 %) > AC-4 (fine speckled, 19.3 %) > AC-5 (large/coarse speckled, 15.2 %) as compared to AC-5 (42.5 %) > AC-1(35 %) > AC-4(22.5 %) in the SP-C+ group; and AC-1(47.1 %) > AC-4(38.2 %) ≫ AC-5(0 %) in the SP-D+ group; and (iv) a higher detection of anti-ribosomal Ab (3x10^−5^<*p* < 0.002). The DP + group was also at risk to develop lupus nephritis (56.8 % versus 2.4 % in SP-C+ and 29.7 % in SP-D+ groups, p < 0.04) and serositis (30 % versus 9.5 % in SP-C+ group, *p* = 0.006) during follow-up (Fig. 3D).

**Table 2:** Characteristics of the three anti-dsDNA/chromatin serological groups from the retrospective cohort of 269 patients with systemic lupus erythematosus (SLE). Data were collected at first occurrence (active phase), except for clinical presentation and therapy response group, which were collected during follow-up.

	DP+	SP-C+	SP-D+	P values DP + vs SP-C+	P values DP + vs SP-D+
Number	190/269 (70.6 %)	42/269 (15.6 %)	37/269 (13.8 %)		
F:M	171:19	36:6	35:2	0.414	0.541
Age, years	31 (24–43)	32 (28–44)	42 (34.5–51)	0.999	0.09
Time from SLE diagnosis, years	3 (0–13)	0 (0–10)	0 (0–9)	0.277	0.339
c-SLEDAI-2K	6 (4–12)	4 (4–6)	6 (4–8)	0.002	0.367
HCQ ± GC	180/184 (97.8 %)	37/42 (88.1 %)	31/37 (83.8 %)	0.012	0.002
IS ± biotherapy	121/184 (65.8 %)	19/42 (45.2 %)	14/37 (37.8 %)	0.021	0.003
IgG anti-dsDNA, IU/mL	110 (33–300)	<10	31 (18–44)	–	4x10^−8^
IgG anti-Chr, AU/mL	8 (7.1–8)	6 (4–8)	<1	–	3x10^−5^
HEp-2 AC-1/4/5/x	119/29/27/3	14/9/17/0	16/13/0/5	2x10^−4^	7x10^−4^
IgG anti-SSA 60 kDa	82/174 (47.1 %)	17/40 (42.5 %)	7/38 (18.4 %)	0.725	0.001
IgG nti-SSA 52 kDa	48/174 (27.6 %)	10/40 (25 %)	6/35 (17.1 %)	0.848	0.289
IgG anti-SSB	28/174 (16.9 %)	7/40 (17.5 %)	4/35 (11.4 %)	0.818	0.612
IgG anti-Sm	91/174 (52.3 %)	22/40 (55 %)	3/35 (8.6 %)	0.861	6x10^−7^
IgG anti-SmRNP	99/174 (56.9 %)	28/40 (70 %)	2/35 (5.7 %)	0.154	4x10^−9^
IgG anti-RNP A	92/174 (52.9 %)	26/40 (65 %)	5/35 (14.3 %)	0.217	2x10^−5^
IgG anti-RNP 68	42/174 (24.1 %)	18/40 (45 %)	0/35 (0 %)	0.011	3x10^−4^
IgG anti-Ribosomal	59/174 (33.9 %)	4/40 (10 %)	1/35 (2.9 %)	0.002	3x10^−5^
IgG anti-CL	40/161 (24.8 %)	9/36 (25 %)	10/33 (30.3 %)	0.690	0.517
IgG anti-β2 GPI	30/149 (20.1 %)	7/34 (20.6 %)	11/33 (33.3 %)	0.999	0.111
**Follow-up from inclusion**					
Follow-up, years	4.6 (2.1–8.3)	4.8 (2.1–8.6)	5.1 (1.8–8.7)	0.878	0.980
Median nb of time points/patient	12	6.8	8	–	–
Therapy response: CR/PR/NR	77/86/27	22/17/3	18/12/7	0.230	0.307
Mucocutaneous	164/190 (86.3 %)	36/42 (85.7 %)	32/37 (86.5 %)	0.999	0.999
Musculoskeletal	166/190 (87.4 %)	35/42 (83.3 %)	30/37 (81.1 %)	0.460	0.302
Hematological	83/190 (43.7 %)	16/42 (38.1 %)	10/37 (27 %)	0.606	0.069
Renal (with PKB)	108/190 (56.8 %)	1/42 (2.4 %)	11/37 (29.7 %)	4x10^−8^	0.036
Serositis	57/190 (30 %)	4/42 (9.5 %)	7/37 (18.9 %)	0.006	0.231
Neuropsychiatric	31/190 (16.3 %)	3/42 (7.1 %)	7/37 (18.9 %)	0.153	0.639

Abbreviations: F: female; M: male; HCQ: hydroxychloroquine; GC: glucocorticoids; IS: immunosuppressant drugs; Chr: chromatin; CL: cardiolipin, β2 GPI: beta 2 glycoprotein I; CR: complete responder; PR: partial or relapse responder; NR: non-responder; PKB: percutaneous kidney biopsy; DP+: double positive dsDNA/Chr group; SP-C+: single positive Chr group; SP-D+: single positive dsDNA group. Statistical analysis: categorical data were analyzed using the Fisher's exact test (2x2), and quantitative data using the non-parametric Mann-Whitney test.

### 3.3 Anti-dsDNA/Chr Ab groups as biomarkers for therapeutic response

Clinical trial results have reported that improvements in the levels of anti-dsDNA/Chr Ab, alongside the achievement of seronegativity in some cases, may serve as a predictive biomarker for therapeutic response [21,22]. Consequently, fluctuations in anti-dsDNA and anti-Chr Ab levels across the three serological groups were assessed at two time points: the inclusion visit (time = 0) and the final visit (time = 1). Given that some patients turn to seronegative during the follow-up period, a survival analysis was also conducted utilizing the log-rank test from T = 0. The criteria for response were defined as follows: complete response (CR) achieved subsequent to the first line of treatment; partial response and/or relapse with drug intensification during follow-up (PR), and a non-response (NR) characterized by a cSLEDAI score of ≥4 during follow-up. Analysis revealed no statistical differences in response distribution among CR, PR, and NR between the DP+, SP-C+, and SP-D+ groups (Table 2).

**Fig. 4:**
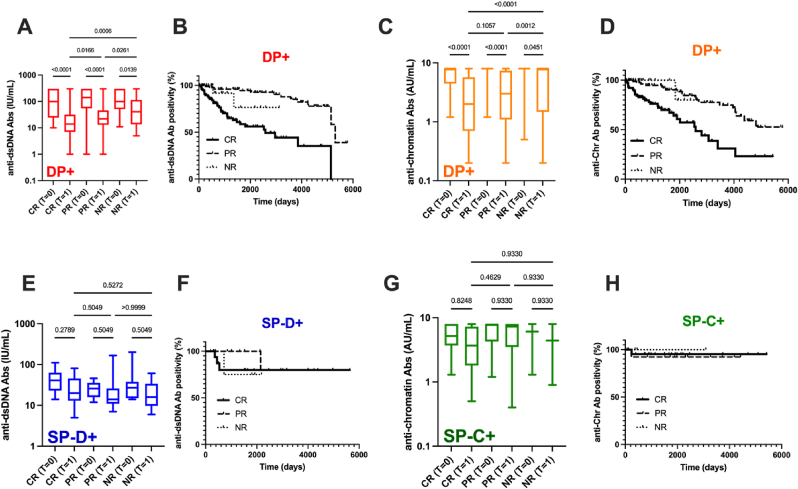
**Anti-dsDNA/Chr Ab groups as biomarkers for therapeutic response.** Anti-dsDNA Ab level variations in the double anti-dsDNA/Chr Ab (DP+) group, according to the therapeutic response, across two time points from inclusion in the follow-up study (time [T] = 0) to the last visit (T = 1) (**A**) or to the first negativity time point plot in a Kaplan-Meier survival curve (**B**). Anti-Chr Ab level variations **(C)** and Kaplan-Meier curve (**D**) in the DP + group. Anti-dsDNA Ab level variations **(E)** and Kaplan-Meier curve (**F**) in the single positive (SP-D+) group. Anti-Chr Ab level variations **(G)** and Kaplan-Meier curve (**H**) in the single positive (SP-C+) group. Abbreviations: CR: complete and stable responders according to the DORIS criteria; PR: partial and/or relapsing responders; NR: non-responders presenting a cSLEDAI ≥4 during follow-up. The p values are reported (Kruskal-Wallis test with Dunn's post-hoc correction).

In the DP + group (Fig. 4A/B), the anti-dsDNA Ab levels decreased in all response groups with a more pronounced effect reported in CR that achieved negativity with a median time of 2587 days versus 5309 days in the PR subgroup and an undefined time in the NR subgroup (*p* = 6x10^−8^). Similar reports were retrieved when regarding anti-Chr Ab in the DP + group (Fig. 4C/D). In contrast, anti-dsDNA and anti-Chr Ab levels in the SP-D+ and SP-C+ groups, respectively, remained stable over time irrespective of the therapeutic response (Fig. 4E–H).

### 3.4 Anti-dsDNA/Chr groups as biomarkers for disease activity, alongside the correlation between anti-dsDNA/Chr Ab and other SLE-associated biomarkers during follow-up

The clinical utility to follow disease activity using anti-dsDNA/Chr Ab levels was studied next across the 3 dsDNA/Chr serological groups. To this end, the repeated-measure correlation (RmCorr) approach was selected to compare intra-individual variations during follow-up between paired anti-dsDNA/Chr Ab level with c-SLEDAI and SLE-associated biomarkers.

**Fig. 5:**
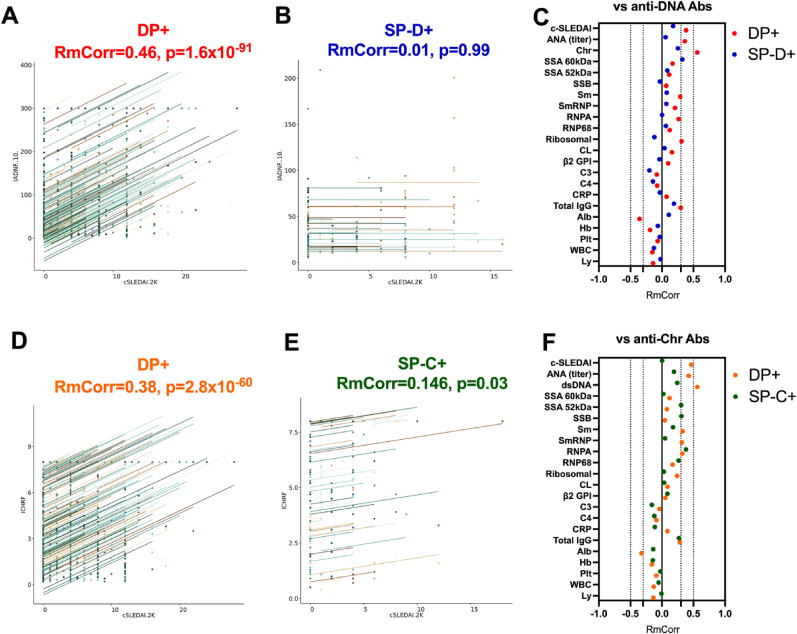
**Anti-dsDNA/Chr Ab groups as biomarkers for assessing disease activity, alongside the correlation between anti-dsDNA/Chr Ab and other SLE-associated biomarkers during follow-up**. **A:** In the double positive dsDNA/Chr (DP+) group, correlation between variations in anti-dsDNA Ab level and the clinical (c)-SLEDAI-2K across all follow-up time points, using a repeated-measure correlation (RmCorr) approach. **B:** In the single positive dsDNA (SP-D+) group, anti-dsDNA Ab level variations versus c-SLEDAI-2K. **C:** Correlation between anti-dsDNA Ab levels versus other SLE-associated biomarkers in the DP+ (red) and SP-D+ (blue) groups. **D:** In the DP + group, anti-Chr Ab versus c-SLEDAI-2K. **E:** In the single positive Chr (SP-C+) group, anti-Chr Ab versus c-SLEDAI-2K. **F:** Correlation between anti-Chr Ab level variations versus SLE-associated biomarkers in the DP+ (orange) and SP-C+ (green) groups.

As presented in Fig. 5A/B, intra-individual anti-dsDNA Ab trajectories, in the DP + group (n = 190 with a median of 12 time points analyzed/patient), presented a correlation with disease activity based on cSLEDAI-2K (RmCorr = 0.46, *p* = 1.6x10^−91^), which was not the case when exploring the SP-D+ group (n = 37 with a median of 8 time points/patient; RmCorr = 0.001, *p* = 0.989). Moreover, with regards to correlations with other SLE-associated biomarkers tested during the follow-up (Fig. 5C), anti-dsDNA Ab trajectories in the DP + group were correlated with the ANA titer on HEp-2 cells (RmCorr = 0.36, p = 4.3x10^−52^), serum albumin (RmCorr = −0.35, *p* = 1.5x10^−41^), and anti-ribosomal Ab level (RmCorr = 0.312, *p* = 5.8x10^−36^). No correlation with RmCorr>(±)0.3 was reported in the SP-D+ group except between anti-dsDNA and anti-SSA 60 kDa Abs (RmCorr = 0.32, *p* = 6.3x10^−6^).

Regarding anti-Chr Ab (Fig. 5D–F), correlation with disease activity was restricted to the DP + group (RmCorr = 0.38, *p* = 2.8x10^−60^) and for this group correlation with SLE-biomarkers included the ANA titer on HEp-2 cells (RmCorr = 0.42, *p* = 6.2x10^−72^) and anti-Sm/SmRNP/RNP-A Ab (0.31<RmCorr<0.33). In the SP-C+ group (n = 42 with a median of 6.8 time points/patient), correlations were retrieved with anti-RNP-A, anti-SSB, and anti-SSA 52 kDa Ab (RmCor = 0.38, 0.31, and 0.30, respectively).

### 3.5 Anti-dsDNA/Chr Ab groups as biomarkers for flares

Previous studies have reported that an increase in anti-dsDNA/Chr Ab levels reflects flares in the course of SLE [23,24]. To better assess these changes depending on the dsDNA/Chr groups, a subset of 140 SLE patients (108 DP+, 13 SP-D+, and 19 SP-C+) was selected based on the availability of serologic data before the onset of a flare (pre-flare group) and at a flare corresponding to clinical worsening with treatment intensification (flare group).

**Fig. 6:**
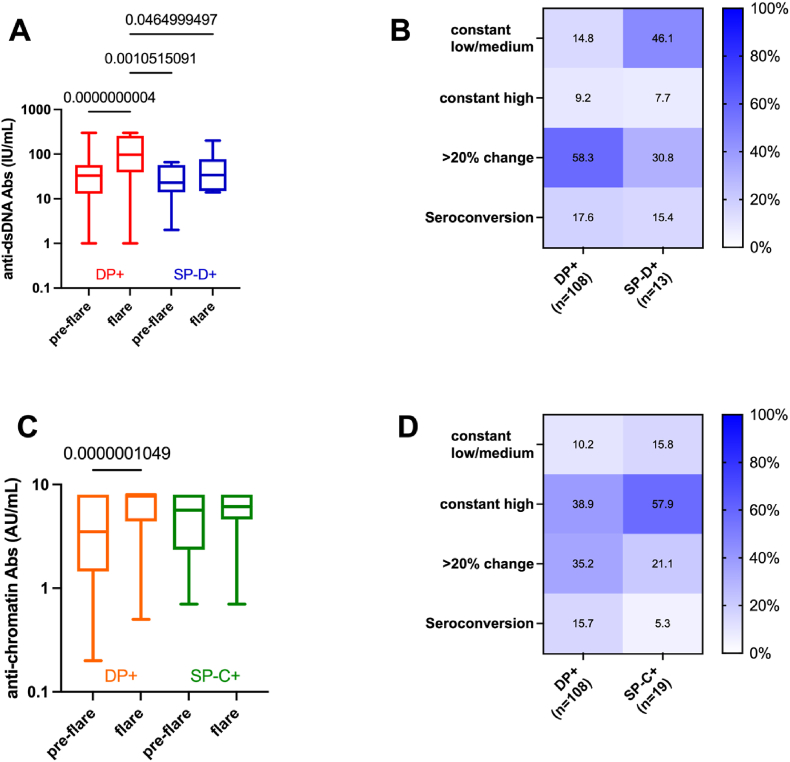
**Anti-dsDNA/Chr Ab groups as biomarkers for flares.** Anti-dsDNA and anti-Chr antibody (Ab) levels were collected from 140 patients with available data prior to flare occurrence (pre-flare) and at flare corresponding to treatment intensification. **A:** Anti-dsDNA Ab levels in the double positive anti-dsDNA/Chr antibody (Ab) group (DP+) and in the single positive anti-dsDNA Ab group (SP-C+). **B:** Anti-dsDNA Ab changes between pre-flare and flare were interpreted as follows: seroconversion corresponding to a shift from a negative to a positive serology (>10 IU/mL); an increase in anti-dsDNA Ab with a change >20 %; less than 20 % change in anti-dsDNA Ab at high levels (arbitrary fixed at > 60 IU/mL); and less than 20 % change in anti-dsDNA Ab at low/medium levels (10–60 IU/mL). **C:** Anti-Chr Ab levels. **D:** Anti-Chr Ab changes between pre-flare and flare (thresholds: positivity >1 AU/mL and high positivity level arbitrary fixed at 4 AU/mL). Significant p values are indicated (Kruskal-Wallis test with Dunn's post-hoc correction).

As reported in Fig. 6, flares were associated in the DP + group, but not in the SP-D+ and SP-C+ groups, with increases in anti-dsDNA and anti-Chr Ab levels (*p* = 4x10^−10^ and *p* = 10^*−7*^, respectively). These changes reflected distinct anti-dsDNA and/or anti-Chr Ab trajectories in the DP + group based on the serological status at the pre-flare stage: seroconversions (17.6 % and 15.7 %, respectively), an increase >20 % (58.3 % and 35.2 %, respectively), a constant elevated level (9.2 % and 38.9 %, respectively), and in a few cases a constant low/medium level (14.8 %, 10.2 %, respectively). Regarding the SP-D+ group, anti-dsDNA Ab level variations from pre-flare to flare stage remained stable in most of the cases (53.8 %) as compared to seroconversions (15.4 %) and an increase >20 % (30.8 %). In the case of the SP-C+ group, a constant elevated level at the pre-flare stage (57.9 %) together with anti-Chr Ab changes (26.4 %) were most frequently associated with flares.

## 4 Discussion

The primary aim of this study was to assess the utility of coupling anti-dsDNA with anti-Chr Ab detection to monitor SLE patients. For that, patients were selected from a cross-sectional cohort and next, SLE patients were analyzed longitudinally. Main results from this study are as follows: (i) in patients with an immune disease compatible with the diagnosis of SLE, a DP + profile strongly supports the diagnosis of SLE compared to a SP-C/D+ profile; (ii) a DP + serological profile established in an active phase of the disease is associated with a more aggressive disease and an elevated risk of developing LN or serositis; (iii) Fluctuation in anti-dsDNA Ab (and anti-Chr Ab) with regard to disease activity is restricted to the DP + serological group, established at the first occurrence, and therefore, it may be used to predict flares and therapeutic response; (iv) A persistent serological activity is retrieved in the single positive SP-C/D+ groups and for them the utility of serial anti-dsDNA/Chr Ab determination to monitor SLE may be limited.

The concept that anti-DNA Ab targets a unique antigenic structure with high specificity in SLE is overstated as the primary immunization is suspected to be directed against Chr with immunization starting at the pre-clinical stage of the disease 2 years before anti-DNA immunization [12,25,26]. Then, the polyclonal anti-DNA Ab spectrum in SLE encompasses a large panel of antigenic targets including distinct DNA structures (e.g., Z and B forms), various DNA origins (e.g., native, plasmid, bacterial, synthetic), single/double stranded DNA, plus phospholipids and cross-reactive proteins at the origin of the important discordances for assays currently in use [27]. As a logical consequence, and confirmed in our cross-sectional analysis, coupling anti-Chr Ab detection with anti-dsDNA Ab is a useful tool in the diagnosis of SLE and best predicts SLE patient evolution [[28], [29], [30]]. Part of the SP-C+ patients can also develop a delayed immunization against dsDNA after SLE classification and for them seroconversion was associated with a severe flare (3 cases reported here). A subset of SLE patients referred to as SP-D+ supports a non-canonical immunization pathway that reacts with free DNA and is most probably driven by a large panel of immune-related conditions including infections and cancers [31].

The first report of anti-Chr Ab in SLE is related to the description in 1947 by M M. Hargraves of the “L.E. factor” that causes opsonization of nuclear material and, half a century later in the 2000s, it was deemed necessary to develop commercial assays with stable antigens, mainly from bovine Chr [32,33]. Meta-analysis has demonstrated that anti-Chr Ab assays performed better in terms of sensitivity and had comparable specificity to anti-dsDNA Ab assays in diagnosing early SLE and drug induced lupus (3 cases in our initial analysis) [11,34]. In patients with LN, elevated anti-Chr Ab levels are reported in those with proliferative LN (class III/IV) at diagnosis and following therapeutic introduction, the anti-Chr Ab (and anti-dsDNA) level declines faster in those with a CR as we report in the DP + group [35]. We and others have established that anti-dsDNA and/or anti-Chr Ab positivity can be used to discriminate cutaneous LE (seronegative) from systemic LE [19,36]. However, anti-Chr Ab detection remains neglected by physicians for two main reasons. First, anti-dsDNA Ab are included in the classification and disease activity criteria for SLE, which is not the case for anti-Chr Ab. Second anti-Chr Ab are specific for SLE but problematic for differentiating SLE (usually at high/medium level) from AID/ID (low level) as reported within the SP-C+ group from the preliminary and cross-sectional analysis [37]. Altogether, this study and others may help in reconsidering the utility of anti-Chr Ab coupled with anti-dsDNA Ab for the classification and follow-up of SLE patients [38].

Through the analysis of Ab subgroups and their longitudinal evolution, more thorough monitoring of SLE patients can be attained. This implies as a prerequisite to establish the serological groups in an active phase of the disease, ideally at diagnosis. To limit misclassification, we have fixed the first occurrence (time = 0) corresponding to diagnosis or flare in our study. Two important organ impairments, LN and serositis, differed between the DNA/Chr groups and this may be explained by the higher prevalence of anti-dsDNA and anti-Chr Ab as already reported by others in these settings [39,40]. Our study retrieved a relatively persistent stable expression in the SP-C+ and SP-D+ groups as compared to the DP + group that fluctuates with disease activity and therapeutic response. Most probably the DP + group may share an elevated IFN type I/II activity to drive dsDNA/Chr Ab levels based on overlap with previously described clusters positive for dsDNA and Chr Abs [39,41,42].

The study presented several limitations. Firstly, the monocentric design of the study and the exclusion of patients with a double negative anti-dsDNA/Chr Ab profile during follow-up limit the generalization of the results to all the spectrum of SLE. Secondly, the retrospective design of the study precluded an extensive characterization of the anti-dsDNA/Chr Ab groups, which needs a better inclusion of clinical parameters such as those established by the BILAG (British Isles Lupus Assessment group index), as well the consideration of additional biological parameters such as genomic variants, cytokines including type 1 and 2 interferon signatures, and cellular subset analysis. Thirdly, the assay used for testing anti-dsDNA/Chr Ab was conducted in a single accredited hospital laboratory utilizing the same instruments and techniques. Consequently, we could not exclude inter-laboratory variations in establishing anti-dsDNA/Chr Ab groups and intra-laboratory variations (batch effect) during the follow-up, which is limited by the use of internal and external quality controls. Fourthly, IgA and IgM isotypes plus IgG subclasses were not considered in our study, which may be clinically pertinent [43,44].

## 5 Conclusion

Our study contributes to a better characterization of SLE patients by proposing that the establishment of the anti-dsDNA/Chr serological groups may help to facilitate the categorization of patients into distinct endotypes. Moreover, discriminating double from single positive anti-dsDNA/Chr Ab patients presents a promising biomarker approach that could enhance prediction regarding organ involvement, disease trajectory, and immunosuppressive therapy evolution. Future and multicentric studies may help to better appreciate the added value of this approach.

## CRediT authorship contribution statement

**Caroline Carlé:** Writing – review & editing, Writing – original draft, Data curation. **Françoise Fortenfant:** Writing – review & editing, Data curation. **Chloé Bost:** Writing – review & editing, Data curation. **Julie Belliere:** Writing – review & editing, Data curation. **Stanislas Faguer:** Writing – review & editing, Data curation. **Dominique Chauveau:** Writing – review & editing, Data curation. **Antoine Huart:** Writing – review & editing, Data curation. **David Ribes:** Writing – review & editing, Data curation. **Laurent Alric:** Writing – review & editing, Data curation. **Gregory Pugnet:** Writing – review & editing, Data curation. **Laurent Sailler:** Writing – original draft, Formal analysis, Data curation, Conceptualization. **Yves Renaudineau:** Writing – review & editing, Writing – original draft, Validation, Resources, Project administration, Methodology, Investigation, Formal analysis, Data curation, Conceptualization.

## Funding

This research did not receive any specific grant from funding agencies in the public, commercial, or not-for-profit sectors.

## Declaration of competing interest

The authors declare that they have no known competing financial interests or personal relationships that could have appeared to influence the work reported in this paper.

## Data Availability

The data underlying this article will be shared following a reasonable request to the corresponding author.
